# The Impact of Overweight and Obesity on Pregnancy: A Narrative Review of Physiological Consequences, Risks and Challenges in Prenatal Care, and Early Intervention Strategies

**DOI:** 10.1007/s11892-025-01585-3

**Published:** 2025-04-21

**Authors:** Kathrin Brunner, Tina Linder, Philipp Klaritsch, Andrea Tura, Karin Windsperger, Christian Göbl

**Affiliations:** 1 Karl Landsteiner Private University for Health Sciences, Krems an der Donau, Austria; 2 Department of Obstetrics and Gynaecology, Division of Obstetrics and Feto-Maternal Medicine, Medical University of Vienna, Vienna, Austria; 3 Department of Obstetrics and Gynaecology, Medical University of Graz, Graz, Austria; 4 CNR Institute of Neuroscience, Padua, Italy

**Keywords:** Overweight, Obesity, Prenatal care, Pregnancy risks, Ultrasound, Intervention strategies, Preconceptional counseling

## Abstract

**Background:**

While substantial literature exists on the intersection of overweight/obesity (OWO) and pregnancy, much of it focuses on specific aspects, making it difficult to maintain an overview of clinically relevant factors for optimal care of OWO women throughout pregnancy.

**Objectives:**

To provide a comprehensive synthesis of the existing literature, covering the full spectrum of clinically relevant information needed to manage OWO women from preconception to birth.

**Methods:**

For this narrative review a literature search was conducted on PubMed in January 2025. Eligible studies included full-text English articles with data from human subjects, with no restrictions on publication date.

**Findings:**

The impact of OWO on pregnancy is multifaceted, encompassing four interrelated themes: physiological consequences, emerging risks, challenges in prenatal care, and intervention strategies. OWO women exhibit differences in metabolic and inflammatory pathways compared to normal-weight women, reflected in altered laboratory tests. When managing gestational diabetes and preeclampsia, obesity-related characteristics must be considered. Clinicians need to be alert of obesity-mediated fetal complications, including overgrowth, malformations, stillbirth, and preterm birth, while navigating challenges in ultrasound measurements. Interventions during the preconception and prenatal periods provide key opportunities to optimize maternal weight and reduce the risk of long-term disease development.

**Conclusion:**

The review’s insights enhance clinical practice and call on researchers and policymakers to prioritize strategies that offer early counseling for obese pregnant women. These initiatives aim to optimize outcomes for both mother and child and contribute to combating the global obesity crisis.

## Introduction

The global prevalence of obesity has significantly increased in recent decades, leading the World Health Organization (WHO) to declare it a “global pandemic” [1, 2]. Currently, approximately 2.5 billion adults worldwide are overweight, 890 million of whom are classified as obese [1]. This represents 44% of women aged 18 and older, and thus includes those in the reproductive age range [1, 3]. In the United States, over 50% of expectant mothers are overweight or obese, whereas in European countries, the prevalence is estimated to range between 7 and 25% [1, 3, 4].

These are alarming data, as the impact of maternal overweight and obesity (OWO) is profound. OWO is not simply the excess accumulation of body fat categorized as overweight (body mass index [BMI] of 25–29.9 kg/m²), obesity (BMI ≥ 30), or severe obesity (BMI ≥ 40) [2, 5, 6]; rather, it is a chronic disease disrupting metabolic pathways, causing insulin resistance and hyperinsulinemia, dyslipidemia, and altered levels of (free) fatty acids, acylcarnitine species, and amino acids, as well as chronic inflammation and oxidative stress [3, 6–11]. Notably, these factors contribute to early placental and fetal dysfunction [3, 4, 6, 8, 12–14].

Hence, pregnancies complicated by OWO face a higher risk of adverse outcomes for both mother and child. Obese mothers are more likely to develop gestational diabetes mellitus (GDM), hypertension, preeclampsia (PE), and birth complications [4, 6, 7]. Fetuses are at risk of overgrowth, congenital anomalies, stillbirth, and preterm birth [3, 4, 6, 10]. Moreover, the impact of maternal OWO extends beyond the fetal period. Evidence indicates that intrauterine exposure to maternal OWO can predispose offspring to long-term health problems in childhood and beyond, potentially creating a cycle of obesity across generations [3, 4, 6, 7, 9].

Breaking this vicious cycle is crucial, ideally before pregnancy, by implementing strategies aimed at addressing OWO [2, 15]. These measures include lifestyle modifications, recommendations regarding gestational weight gain (GWG), and, in some cases, pre-conceptional pharmaceutical intervention [2, 16]. In cases of severe obesity, bariatric surgery (BS) has been shown to be an effective intervention for long-term weight reduction [2, 17–19].

Overall, the intersection of OWO and pregnancy represents a complex but compelling field of study that has garnered significant attention in recent years [3]. Since 2000, nearly 25,000 PubMed citations (search terms “obesity” and “pregnancy”) have been generated. Numerous large cohort studies, systematic reviews, and meta-analyses have attempted to explore the topic in detail (e.g., 3, 4, 6, 8, 18, 20, 21), yet they predominantly focus on specific aspects of the broader issue. Consequently, maintaining an overview of the clinically relevant topics amid this vast body of literature is challenging, making it difficult to provide optimal care for OWO women throughout their pregnancies.

This narrative review seeks to bridge the research gap by providing a comprehensive synthesis of the existing literature, covering the full spectrum of clinically relevant information needed to manage OWO women from preconception to birth. Through a critical analysis of recent studies, the review offers a nuanced understanding of the physiological consequences in pregnancies complicated by OWO and the emerging risks for both mother and child. It also addresses challenges in the prenatal care of OWO women and outlines early intervention strategies. The goal is to emphasize the multifaceted nature of prenatal care in the context of OWO, identify gaps in current knowledge, and propose directions for future research, ultimately contributing to improved clinical outcomes for both women and their offspring.

## Methods

### Search Strategy and Selection Criteria

A narrative review was conducted to assess the impact of OWO on clinically relevant aspects of pregnancy care. An electronic literature search was performed in January 2025 using the PubMed database with the following MeSH terms and Boolean operators: “pregnancy or gestation and overweight or obesity,” “gestational weight gain,” “pregnancy and hyperinsulinemia,” “pregnancy and insulin sensitivity,” “insulin resistance,” “placenta,” “placental changes,” “prenatal imaging,” “obesity and prenatal diagnostics,” “fetal growth,” “fetal macrosomia,” “obesity and gestational diabetes,” and “obesity and bariatric bypass surgery or gastric bypass.” Eligible studies included full-text English articles with human subject data, without restrictions on publication date. We prioritized meta-analyses, systematic reviews, RCTs, longitudinal observational studies, and cohort studies. To ensure high-quality, peer-reviewed content, grey literature was excluded from the analysis.

### Screening, Data Extraction Process, and Quality Assessment

The procedure followed in this narrative review included the identification of relevant literature, selection, extraction, and compilation of thematic themes [20]. Two reviewers independently conducted literature searches, initially screening titles and abstracts to assess the scope and relevance to the research aim. All articles identified in this first step were imported into EndNote, a reference management software. Next, a full-text screening was conducted to apply inclusion and exclusion criteria to the selected articles. Data were then extracted using a standardized protocol in Microsoft Excel. The extracted data included authors, year of publication, BMI categories, physiological consequences, prenatal care risks, challenges in prenatal care, and early intervention strategies. Articles reviewed by one author were cross-checked by the other for accuracy and completeness. The themes incorporated into the narrative review were those deemed most relevant to current clinical practice, according to the authors. Data collection continued until saturation was reached [21]. To evaluate the methodological quality of the included studies, we used the open-access tool “Assessment Scale for Narrative Reviews (SANRA),” as recommended by Baethge et al., who encourage its use for critically appraising articles while preparing narrative reviews [22]. Any discrepancies were resolved through team consensus. As most of the papers we reviewed did not differentiate between sex and gender identity, we have maintained this approach in our narrative review for clarity and consistency. Therefore, terms such as ‘women’ and derivatives are used throughout the manuscript.

## Physiological Consequences of OWO in Pregnancy

Pregnancy is a unique metabolic state, as significant metabolic adaptations are necessary to ensure adequate growth and development of the fetus while meeting the increased energy demands of the mother [3, 6, 23]. Although this metabolic balance throughout normally progressing pregnancies is well understood, knowledge about the impact of OWO on different metabolic adaptations is still evolving. Evidence to date shows that, compared to normal weight women, the metabolic alterations in OWO women occur in key areas, including fat accumulation, insulin resistance and hyperinsulinemia, dyslipidemia, altered levels of (free) fatty acids, acylcarnitine species, and amino acids, as well as chronic inflammation and oxidative stress [3, 6, 8, 11, 24–26].

### Fat Accumulation

An increase in maternal abdominal adipose tissue—an endocrine-active tissue generated by adipocytes—is an important adaptive response during pregnancy. Generally, adipose tissue can be stored in two primary depots: visceral adipose tissue (VAT) and subcutaneous adipose tissue (SCAT). VAT, which surrounds abdominal organs, differs from SCAT in its endocrine, lipolytic, and immune functions, playing a significant role in metabolic and inflammatory responses related to adiposity [27]. Several studies suggest that abdominal adipose tissue is preferentially deposited in the visceral compartment as gestation progresses; however, significant differences in fat deposition and mobilization across BMI categories have been found [28–33]. For example, Straughen et al. showed through ultrasound measurements that preperitoneal fat thickness (pmax) increases, while subcutaneous fat thickness (smin) decreases in normal-weight women as pregnancy advances [28]. In contrast, OWO women have larger anatomical depots of adipose tissue in both compartments at all stages of pregnancy; however, both pmax and smin decrease as gestation progresses [28]. The cumulative fat index (pmax plus smin) does not change across pregnancy in normal-weight women, while it decreases in obese women [28]. Clinically, it is frequently observed that OWO individuals gain weight at a slower rate than their non-overweight counterparts [3, 28]. Recent studies using magnetic resonance imaging (MRI) have further confirmed altered changes in visceral adipose tissue stores in pregnant women that correspond to BMI categories [33, 34].

These data highlighting differences between OWO and normal-weight women in fat accumulation during pregnancy may explain the altered metabolic adaptations associated with OWO [28, 33]. Hence, there is a pressing need for further investigations concerning the recently evolving evidence that the metrics VAT and SCAT may be superior to BMI in predicting maternal risks, findings that would guide future clinical practice [33, 35].

### Insulin Resistance and Hyperinsulinemia

Insulin resistance, induced by placental hormones, contributes to the physiological diabetogenic effect of pregnancy on maternal metabolism [3, 36]. Various studies report that reductions in insulin sensitivity range from 40 to 80%, with a more pronounced effect observed in OWO women compared to those of normal weight [3, 10, 36, 37]. In this context, obesity is associated with a lower insulin sensitivity, and higher homeostasis model assessment of insulin resistance scores—HOMA-IR and HOMA2-IR [25, 38, 39]. As a compensatory response, beta cells increase insulin secretion, with studies indicating a higher insulin release in obese pregnant women compared to those of normal weight [3, 25, 40]. However, Linder et al. demonstrated that, unlike in normal-weight women, the increase in insulin release in obese women is insufficient to fully compensate for the decrease in insulin sensitivity in pregnancy [11]. This is reflected in a lower disposition index associated with elevated BMI, suggesting impaired beta-cell function in mothers with obesity [11]. Similarly, OWO is closely linked to further alterations in glucose metabolism parameters, such as HbA1c, fasting glucose, and C-peptide concentrations [3, 10, 11, 41]. The maternal metabolic environment of insulin resistance and hyperinsulinemia in obese women affects early placental growth, gene expression, and function, which may manifest clinically as pregnancy progresses [3, 8, 42]. For example, obesity was shown to be associated with higher placental weight at birth, placental vascular dysfunction, placental inflammation, and alterations in placental transporter and mitochondrial activity [8, 42].

### Dyslipidemia

Maternal hyperlipidemia develops physiologically with increasing gestational age, peaking in the third trimester when fetal growth accelerates. Specifically, total cholesterol (TC), triglycerides, high-density lipoprotein cholesterol (HDL-C), and low-density lipoprotein cholesterol (LDL-C) are elevated in pregnant women compared to nonpregnant women, with variations depending on ethnicity [6, 43]. In the presence of OWO, however, lipid levels can surpass the physiological range of increase [6]. In OWO women, significantly higher concentrations of TC and LDL-C are observed compared to their normal-weight counterparts [6]. Studies focusing on the differential aspects of OWO on lipid metabolism reported higher TC and LDL-C levels at baseline, but a less pronounced increase as pregnancy progressed in OWO compared to normal-weight women [44, 45]. Furthermore, Stadler et al. reported lower HDL-C, an increase in HDL cholesterol efflux capacity, and a decrease in the activity of lecithin-cholesteryl acyltransferase (LCAT)—an enzyme important for HDL maturation—in mothers with OWO compared to pregnant women with normal weight [46]. Some studies have found a BMI-associated increase in triglycerides, while others have not [6, 10, 45, 47]. Additionally, early pregnancy levels of ApoB (apolipoprotein B) and the ApoB/ApoA (apolipoprotein A) ratio have been positively associated with pre-pregnancy BMI [6, 48]. To differentiate between physiological hyperlipidemia and obesity-related pathological dyslipidemia, reference values have been proposed by several authors since the 1990s [49, 50]. However, these have not yet been incorporated into clinical practice.

### Altered Levels of (Free) Fatty Acids, Acylcarnitine Species, and Amino Acids

A surplus of body fat combined with insulin resistance contributes to increased lipolysis, resulting in elevated fatty acid concentrations [3, 6]. It has been shown that pre-pregnancy BMI is positively associated with the levels of both free fatty acids (FFAs) and fatty acids within glycerophospholipids throughout gestation [3, 6]. Excessive secretion of FFAs may contribute to ectopic lipid accumulation and lipotoxic effects on various organs, including the liver, muscle, heart, and placenta [6, 51]. Specifically, Hellmuth et al. identified increased levels of non-esterified saturated and monounsaturated fatty acids (MUFAs), as well as elevated ratios of palmitoleic to palmitic and oleic to stearic acids, in obese pregnant women compared to their normal-weight counterparts [47]. Regarding polyunsaturated fatty acids (PUFAs), a selective increase in the pro-inflammatory omega-6 PUFAs dihomo-gamma-linoleic acid (DGLA), arachidonic acid, and adrenic acid was observed in OWO women. In contrast, the omega-3 PUFAs α-linolenic acid and eicosapentaenoic acid (EPA) were reduced in women with an obese pre-pregnancy BMI compared to those with a normal pre-pregnancy BMI [6, 9]. As the balance between omega-3 and omega-6 PUFAs is crucial for maintaining a healthy equilibrium between pro- and anti-inflammatory effects, these findings further support the idea that OWO contributes to low-grade systemic inflammation during pregnancy [6, 8, 9].

Data are scarce on the impact of OWO on acetylcarnitine species, which are required for transporting fatty acids into the mitochondria for β-oxidation and energy production [6, 26, 52]. Ryckmann et al. identified higher levels of C2 (acetylcarnitine), C4-OH (3-hydroxybutyrylcarnitine), and C18:1 (oleoylcarnitine) in obese women compared to normal-weight women, particularly in the third trimester of pregnancy [26]. In their longitudinal study, Ryckmann et al. investigated amino acid concentrations in obese versus normal-weight pregnant women and found that only arginine levels were elevated in obese women during the second trimester compared to their normal-weight counterparts [26]. No other significant changes in amino acids were observed between lean and obese women [3, 26, 52].

### Chronic Inflammation and Oxidative Stress

A hallmark of OWO is chronic, low-grade inflammation which disrupts the balance between pro- and anti-inflammatory cytokines essential for placental development and function [8, 13, 24]. This disturbance is reflected in altered biomarkers, with elevated levels of C-reactive protein (CRP) and adenosine deaminase (ADA) in OWO pregnant women compared to those of normal weight [3, 25, 53]. Studies showed a positive correlation between CRP and BMI (a 5-unit increase in BMI resulted in a 46% increase in serum CRP) [38, 53–55], only Christian et al. found a non-significant increase in CRP levels with obesity [56]. Similarly, IL-6 levels were higher in obese women [8, 25, 53]. Some studies, but not all, have linked inflammatory biomarkers to fetal, neonatal, and long-term adiposity outcomes [3, 4, 52, 53]. To better understand inflammation during pregnancy, further research is needed to establish reference levels of serum ADA, CRP, and IL-6 in both OWO and normal-weight pregnancies. The inflammatory environment in OWO promotes reactive oxygen species (ROS), with both mechanisms contributing to adverse pregnancy outcomes [3, 8, 53]. Research into natural products with anti-inflammatory and antioxidant properties (e.g., phenolic acids, punicalagin, and curcumin) offers potential for mitigating OWO-mediated inflammation and oxidative stress [9].

In conclusion, metabolic disruptions in obese women create multiple risk factors for complications in the fetus, mother, and offspring. Given this situation from the start of pregnancy, obese women should be considered high risk and receive enhanced prenatal care [8, 10, 11].

## Risks of OWO in Maternal Care

### Gestational Diabetes Mellitus (GDM)

In 65–75% of cases, pregnant women with GDM are overweight or obese [57, 58]. Linder et al. found that a high pre-gestational BMI was significantly linked to an earlier GDM diagnosis, with obese GDM patients experiencing a greater increase in fasting plasma glucose levels compared to their normal-weight counterparts, although post-glucose load glucose concentrations during the OGTT were similar between the groups [59]. Additionally, GDM patients with OWO require more glucose-lowering medication, with obese women needing the highest daily insulin doses [59–61]. Metformin was used more frequently in obese GDM patients, with higher doses required compared to normal-weight women [59]. Obesity also led to more frequent combined treatment with metformin and insulin [59]. In this context, evidence suggests that metformin monotherapy has a higher failure rate in pregnant women with higher BMI [62–64] and higher fasting glucose levels [63, 64].

Further, it is evident that the impact of maternal OWO on the risk of fetal growth excess is aggravated by hyperglycemia [65]. Linder et al. found this association to be strongest in obese GDM patients requiring glucose-lowering therapy [59]. Hence, GDM combined with obesity is strongly linked to a higher risk of adverse outcomes, including lower Apgar scores, cesarean sections, and neonatal complications requiring hospitalization [59, 66].

Women with a history of GDM are at a significantly higher risk of developing type 2 diabetes and experiencing gestational insulin resistance and hyperglycemia in subsequent pregnancies [67]. The recurrence rate of GDM is approximately 50%, influenced by factors such as elevated BMI, insulin use in previous pregnancies, macrosomic offspring, and laboratory results such as fasting glucose and HbA1c [67, 68].

### Preeclampsia (PE)

Maternal obesity is a well-known risk factor for the development of PE [3, 4, 16, 36, 52, 69–72], with placental mitochondrial dysfunction leading to OS as a likely key pathophysiological mechanism [3, 4, 8, 69]. Xiang et al. identified several adiposity indicators in early pregnancy associated with PE, including pre-pregnancy BMI, VAT, SCAT, waist circumference, waist-to-hip ratio, and GWG [70]. While aspirin is a proven intervention for reducing PE risk, the optimal dosage remains controversial [71–74]. Emerging evidence indicates that OWO patients may require a higher dose of 162 mg for effectiveness [74, 75]. Additional interventions to reduce PE risk in obese women include managing GWG and BS [3, 76]. For PE diagnostics, the sFlt-1/PlGF ratio is well established [77]. While some studies have found an inverse correlation between BMI and sFlt-1, no research has proposed alternative threshold values with sufficient evidence [55, 78, 79]. Therefore, the sFlt-1/PlGF ratio remains a valid tool for diagnosing PE in obese pregnant women [78].

## Risks of OWO in Fetal Care

### Fetal Overgrowth: Macrosomia and Large for Gestational Age (LGA)

OWO mothers have an increased risk of giving birth to infants with “overgrowth”. Most authors define macrosomia as birth weight ≥ 4000 g [3, 80]; others use 4500 g as the cut-off point, irrespective of gestational age [3, 81]. Based on these variations Gaudet et al. proposed categorizing macrosomia into classes ranging from Class I (4000–4499 g) over Class II (4500–4999 g) to Class III (≥ 5000 g) [81]. LGA is defined as a birth weight above the 90th percentile, corrected for gestational age [3, 4, 80]. Heiskanen et al. identified preexisting diabetes, previous macrosomic birth, pregnancy ≥ 42 weeks gestation, maternal pre-pregnancy BMI ≥ 25, fetal male sex, GDM, and nonsmoking as the most relevant risk factors for developing macrosomia [82]. Although fetal growth is regulated by a range of maternal and fetal factors, its association with maternal OWO is likely to arise from increased placental insulin resistance and dysfunction in terms of greater nutrient transfer to the fetus [3, 83, 84]. Accordingly, neonates of obese women are found to be significantly heavier at birth than those of normal-weight women due to an increase in body fat, with both pre-pregnancy maternal BMI and placental weight being the strongest determinants [3, 4, 10].

### Fetal Malformations

Maternal obesity is associated with an increased risk of fetal anomalies [85, 86]. Authors demonstrated that the risk of developing anencephaly, encephalocele, or spina bifida, is significantly higher in obese women compared to normal or overweight women [87–89]. Folic acid supplementation prevents neural tube defects in conditions unrelated to obesity, but these defects persist in obese women, even with a folic acid-fortified diet [16, 87, 89].

Studies investigating metabolic disorders and the risk of congenital heart defects (CHD) found a marginally increased risk for CHDs in women with obesity, GDM, and hypertension, and a strong association between CHDs and pre-gestational diabetes and early-onset PE [3, 4, 52, 85, 90]. Persson et al. demonstrated that the risk of CHD progressively increased with BMI from overweight to severe obesity [85]. Additionally, maternal severe obesity is strongly associated with an increased risk of cleft palate, with or without cleft lip, in offspring [3, 4, 52, 91].

### Stillbirth

An elevated BMI is strongly associated with an increased risk of stillbirth [3, 52, 92, 93]. Evidence indicates that the risk of stillbirth is 1.5 times higher for women with severe obesity compared to those with overweight. Specifically, there is a direct correlation between BMI and the risk of stillbirth at every stage of gestation, with the risk being most pronounced at term [92, 93]. Notably, a BMI of ≥ 50 kg/m² carries a 5.7-fold higher risk of stillbirth at 39 weeks’ gestation and a 13.6-fold higher risk at 41 weeks’ gestation, compared to other BMI categories [92]. Finally, obesity is associated with nearly 25% of stillbirths occurring between 37 and 42 weeks of gestation [92].

### Preterm Birth

Due to the aforementioned maternal and fetal complications, obese women have an elevated risk for idiopathic preterm deliveries, but also for spontaneous preterm labors [3, 4, 52]. Although the pathophysiology of preterm birth in the presence of obesity is not yet well characterized, obesity-associated inflammation is undoubtedly a key factor [3, 8].

## Challenges in Prenatal Care in the Presence of Maternal Obesity

Prenatal imaging, specifically transabdominal ultrasound, plays a significant role in assessing fetal well-being; however, it faces several challenges in the presence of obesity [94–97]. As the additional fatty tissue increases the distance that ultrasound waves must travel to reach the target structures, a reduction in wave energy upon arrival at the inspected structures results. Hence, greater absorption and dispersion of the waves occurs, compromising image quality and raising the likelihood of undetected fetal anomalies [95, 98–101]. Although the association between OWO and low imaging quality is apparent, large-scale studies are scarce. Hildebrand et al. demonstrated that the detection rate of anomalies with long-term handicaps in a routine ultrasound in the first or second trimester was lower in the obese group (27.3%) compared to normal-weight women (46.3%) [98]. Using an extended image dataset acquired by a large number of sonographers on multiple ultrasound machines, Yaqub et al. demonstrated that the higher the maternal BMI category, the less likely fetal images were to satisfy quality criteria [96]. In particular, the poor visibility of the cavum septi pellucidi—a marker for normal brain development that is closely associated with the formation of the corpus callosum—and the findings that head, abdomen, and femur views—importantly relevant for fetal biometry—are affected by the BMI category raise important clinical concerns [96]. Hence, obese women attending routine fetal anatomy scans must be informed about the limitations of imaging [96].

Several techniques have been suggested to overcome ultrasound-related challenges in OWO women [16, 97, 102–104]. Puissegur et al. demonstrated that lowering the emission frequency improves wave penetration, thereby enhancing the accuracy and completeness of fetal anatomical assessments in OWO [104]. Alternatively, transvaginal ultrasound can be utilized during the second trimester, typically in weeks 20–23 of pregnancy, to improve the visualization of fetal structures and facilitate the detection of malformations [103]. Furthermore, scanning through specific anatomical regions, offering an acoustic window that includes the umbilicus, the suprapubic area, and both iliac regions, may optimize the visualization of fetal structures located in proximity to these areas [94].

## Early Intervention Strategies

### Pre-conception

As part of preconceptional obesity management, alongside lifestyle interventions, glucagon-like peptide-1 receptor agonists (GLP-1-RAs)—such as liraglutide and semaglutide—are recommended as the first-line treatment for obese women of reproductive age, provided they are discontinued at least two months prior to conception [2, 16, 18, 52, 105–107]. However, currently data are limited for clinicians to effectively counsel patients regarding accidental periconceptional exposure to GLP-1-RAs [106, 107]. Notably, animal studies have shown adverse outcomes in offspring exposed to GLP-1-RAs, including decreased fetal growth, skeletal and visceral anomalies, and embryonic death [106]. Although large, prospective studies in humans are lacking, emerging data do not indicate a consistent pattern of congenital anomalies or pregnancy losses [106, 107]. At present, patients should be advised that there is insufficient evidence to predict the potential adverse effects, or the absence thereof, in cases of periconceptional exposure to GLP-1-RAs. Therefore, all patients undergoing therapy with GLP-1-RAs are advised to use contraception to prevent unintended pregnancies [18, 106]. Another FDA-approved option is Contrave, a combination of naltrexone and bupropion, although it typically results in less weight loss [18].

BS, an established treatment for severe obesity, particularly when associated with co-morbidities, offers significant benefits for obese patients but also poses potential risks during pregnancy [2, 16, 18, 19, 108, 109]. As the most effective method for achieving long-term weight loss, BS helps to reduce many obesity-related pregnancy complications, such as GDM, PE, and fetal macrosomia [108, 110]. In addition, pregnant women who have undergone BS exhibit altered lipid metabolite profiles, with lower levels of total cholesterol, LDL-C, and triglycerides, compared to obese women without prior surgical intervention. Furthermore, ultrasensitive CRP levels were lower in BS mothers than in obese women, approaching the levels observed in lean controls [17, 108, 111, 112].

However, pregnancy after BS may be associated with maternal nutritional deficiencies, anemia, and an increased risk of small-for-gestational-age infants, prematurity, neural tube defects, or stillbirths [17, 108, 110]. Although the incidence of GDM is lower post-surgery, glucometabolic abnormalities may persist, with BS patients often experiencing postprandial hyperglycemia followed by hypoglycemia two to three hours after glucose intake (dumping syndrome) [108, 112, 113]. As the risks associated with BS vary by technique, further studies are needed to determine the most suitable surgical options for obese women planning pregnancy [108]. Preconception care should ideally focus on delaying pregnancy for at least 12 months after surgery and evaluating micronutrient status [110]. Pregnant women following BS should be screened for GDM using self-monitoring of blood glucose or continuous glucose measurements, as the oral glucose tolerance test is not recommended [19, 110, 114]. Prenatal care must include ongoing nutritional monitoring and supplementation according to international guidelines [19, 114, 115], as well as counseling regarding the risks of surgical complications, such as internal hernia or band slippage [110].

### Post-conception

Although pregnancy, along with preconception care, is a key life stage to adopt healthy lifestyle habits intervention options in this period remain limited [2, 16, 52, 116]. Evidence shows that a structured prenatal diet combined with physical activity is the most effective approach to reduce GWG and improve maternal and neonatal outcomes [2, 16, 52, 117]. Specifically, diet al.one— which has a greater impact on GWG—appears to be associated with both improved maternal and neonatal outcomes, while physical activity alone is primarily linked to better maternal health [16, 116]. The overall goal is to limit GWG to 5–9 kg for obese women, while for overweight women, a GWG of 6.8–11.3 kg is recommended [118]. Ideally, pregnant women should be referred for nutritional counseling and encouraged to engage in physical activity -in the absence of contraindications- according to (inter)national guidelines and recommendations [117, 119, 120]. The findings of several meta-analyses and reviews emphasize the urgent need to implement prenatal lifestyle interventions as part of routine antenatal care [116, 121].

## Discussion

This narrative review examines the impact of OWO on pregnancy care, categorizing it into four interrelated themes: physiological consequences, risks, challenges in prenatal care, and early intervention strategies. Each theme provides clinically relevant information and spans the entire period, from preconception to birth. Unlike other reviews that focus on specific aspects in detail [3, 4, 6, 18, 24, 81], this synthesis offers a comprehensive and up-to-date guide to optimizing pregnancy care in the context of OWO.

It is crucial to note that the effects of OWO extend beyond pregnancy itself [3, 4, 6, 52]. Most interventions aimed at mitigating the obesogenic effects on both mother and fetus typically begin within or after the first trimester, by which time the fetoplacental unit has already been exposed to an adverse metabolic environment [8, 10]. Preliminary data have identified changes in placental gene expression and function before phenotypic changes occur [122]. Therefore, optimizing the metabolic environment—addressing factors such as fat accumulation, insulin resistance, hyperinsulinemia, dyslipidemia, altered fatty acids, acylcarnitine species, amino acids, chronic inflammation, and oxidative stress—before conception is essential [3, 6–10, 52, 116]. For women with OWO planning a pregnancy, preconception counseling and early interventions should be prioritized. Options such as GLP-1-RAs and BS hold considerable potential [2, 16, 18, 19]. Notably, achieving non-obese metabolic status is not necessary; even a 5% weight reduction has been shown to positively influence obesogenic effects on pregnancy [123].

While this review provides a thorough synthesis of knowledge on OWO and pregnancy care, it does not address obstetric challenges that may arise during labor (e.g., failed trial of labor, or prolonged labor) or in the postpartum period (e.g., endometritis, or venous thromboembolism), as these fall outside its scope. Clinicians, however, must remain vigilant in managing these concerns, which are discussed more extensively by other authors, when handling birth care [3, 4].

Importantly, the impact of maternal OWO persists after birth [2–4, 6, 52, 124]. OWO during pregnancy increases the risk of postpartum weight retention and long-term diseases in the mother, including type 2 diabetes and cardiovascular disease [2–4, 6, 124]. Intrauterine exposure to maternal OWO increases the likelihood of obesity and related metabolic and cardiovascular diseases in child- and adulthood [2–4, 6, 12, 52, 124, 125]. To break this intergenerational cycle of obesity, maternal OWO during pregnancy must be recognized not just as an individual concern but as a broader societal and public health issue. Integrating risk assessment tools, recommendations, and early intervention strategies into clinical practice in a cost-effective and accessible manner is essential for maximizing maternal and child health across all social classes [2].

Despite its strengths, this narrative review has limitations. Unlike systematic reviews or meta-analyses, narrative reviews involve subjective study selection and weighting, which can introduce bias in data presentation [20]. Additionally, the variability in research methodologies across studies may limit the generalizability of the findings. Lastly, while the review explores associations between maternal OWO and pregnancy, it cannot establish causality due to the observational nature of most of the included studies.

## Conclusion

This narrative review highlights the multifaceted impact of maternal OWO on prenatal care. As the prevalence of obesity—also among women of reproductive age—continues to reach pandemic proportions, it becomes critical to focus on the preconception and prenatal periods as key opportunities for optimizing maternal weight. The review’s insights not only enhance clinical practice but also urge researchers and policymakers to prioritize strategies that support early counseling for obese women. These efforts aim to promote the best outcomes for both mother and child, disrupt the intergenerational cycle of OWO, and help tackle the global health crisis of obesity.

## Data Availability

No datasets were generated or analysed during the current study.
